# Lateral perforator flap: A surgically and oncologically safe method for patients with breast cancer

**DOI:** 10.1016/j.jpra.2026.05.023

**Published:** 2026-05-17

**Authors:** Laila Shakhtour, Linda Hartman, Emma Niméus, Tor Svensjö

**Affiliations:** a Department of Surgery, Kristianstad Central Hospital, Kristianstad, Sweden; b Department of Clinical Sciences Lund, Division of Surgery, Lund University, Lund, Sweden; c Centre for Mathematical Sciences, Lund University, Sweden; d Department of Surgery, Skåne University Hospital, Malmö, Sweden

**Keywords:** Primary breast cancer, Oncoplastic breast surgery, Perforator flap, Sweden

## Abstract

**Background:**

Chestwall perforator flaps have been used in breast conservation after wide local excision for more than a decade. Although the technique is promising, it still needs to be standardized. For example, no published studies have numerically proven that the operated breast retains its features after operation and radiation. This study compared preoperative- and 1-year postoperative measurements to investigate whether there are any significant changes to the breast.

**Methods:**

The study examined all patients with breast cancer who were operated on with a lateral chest wall perforator flap in a primary setting between the years 2011 and 2021 at the surgical department of Kristianstad Central Hospital. Volume, ptosis, and the sternal notch-nipple distance were measured by the clinician, and standardized photos were analysed with BCCT.core software to determine the cosmetic outcome. A Likert scale of 1–10 was used to subjectively measure cosmetic outcomes.

**Results:**

139 patients diagnosed with breast cancer or DCIS were operated on with breast-conserving surgery and a lateral perforator flap during the study period. For statistical analysis the untreated breast served as the patient’s own control. No difference was found in regard to volume, but the sternal notch-nipple distance and ptosis decreased slightly but significantly by 6 and 5 mm,resepectively (*p* < 0.001). BCCT.core classified the postoperative results as 80% excellent or good, while the preoperative rate was 89%.

**Conclusions:**

This study shows that lateral chest wall perforator flaps are a surgically and oncologically safe technique that conserves common features of the breast according to measurements before and 1 year after operation and radiotherapy.

## Background

Breast-conserving surgery (BCS) followed by radiation therapy is considered the standard of care for most women with early-stage breast cancer.^1^^,^^2^ Studies suggest that breast conservation leads to higher quality of life and satisfaction compared to mastectomy.^3^^,^^4^

Oncoplastic breast surgery combines oncological principles with plastic surgical techniques to achieve both complete tumour excision and favourable aesthetic outcomes. One example of this approach is volume replacement using perforator flaps.^5^, ^6^, ^7^, ^8^, ^9^, ^10^ Local perforator flaps are fasciocutaneous flaps based on cutaneous perforators, that allow immediate partial breast reconstruction for women with small to moderate breast size or larger tumours, thereby avoiding mastectomy.

A key advantage of perforator flaps is the preservation of underlying muscle, which reduces donor site morbidity. Additionally, the main artery pedicle of the chest wall remains intact, which enables a late reconstruction with techniques such as a Latissimus dorsi flap. This study focuses on lateral perforator flaps, which involve using perforators from the lateral chest wall. This technique is well described and increasingly adopted internationally, but further studies are needed to support its broader acceptance as a standard oncoplastic option. At the breast surgery unit in Kristianstad, Sweden, perforator flap reconstruction has been in clinical use since 2011. The aim of this study is to describe a cohort that has undergone operation with a perforator flap during a 10-year period and present follow-up outcomes.

## Methods

This retrospective study included all patients who underwent lateral perforator flap reconstruction in a primary setting between 2011 and 2021 at the Surgical Department of Kristianstad Central Hospital, Sweden. The hospital is located in Southern Sweden and manages approximately 300 new breast cancer cases annually. Eligible patients were diagnosed with primary invasive or non-invasive breast cancer.

Data were retrieved from the operative record system Orbit5™ (SYSteam Health & Care, Huskvarna, Sweden) and the patient record system Melior™ (Siemens Healthcare, Upplands Väsby, Sweden). All data were de-identified and stored in a secure database using REDCap. Statistical analyses were performed using IBM SPSS Statistics for Windows. Descriptive statistics were used to summarize cohort characteristics and Student’s *t*-test was used to assess changes in pre- and postoperative breast dimensions.

The examined variables included patient demographics (age, body mass index (BMI), smoking status, comorbidities), tumour characteristics, and adjuvant therapy. Breast measurements were available for most patients and included:

• breast volume (measured with plastic cups, in milliliters.^11^)
• distance from the sternal notch to the nipple (SNN) (in centimetres)
• ptosis (in centimetres)

Measurements were recorded at diagnosis and at the 1-year follow-up and standardized photographs (frontal and lateral views) were taken at both time points. Notably, both breasts were measured pre- and postoperatively, which allowed each patient to serve as their own control for postoperative changes in the untreated breast.

At the 1-year follow-up, patients rated their satisfaction with the aesthetic result on a 10-point Likert scale (1 = worst, 10 = best). Objective cosmetic evaluation was performed using BCCT.core software.^12^, which grades cosmesis as 1 (excellent), 2 (good), 3 (fair), or 4 (poor) based on symmetry, scar visibility, and colour. The Breast Retraction Assessment (BRA).^13^ was also retrieved from the BCCT.core analysis to quantify breast retraction.

Postoperative complications were recorded and classified according to the Clavien-Dindo system.^14^ For infections, culture confirmation and antibiotic treatment were noted. All patients received adjuvant therapy according to national guidelines. Ethical approval was obtained from the Regional Ethics Review Board of Stockholm, Sweden (2021–04,506).

### Surgical approach

Perforators were identified preoperatively using a handheld Doppler with the patient in a simulated intraoperative position. Doppler was used only prior to surgery and not intraoperatively. Most flaps were based on the LICAP (lateral intercostal artery perforator) vessels, which are typically located approximately 3 cm anterior to the lateral edge of the latissimus dorsi muscle. The LTAP (lateral thoracic artery perforator) was often identified and marked along the anterior axillary line. LTAP flaps are a good option when a more distant breast location requires coverage or when a skin island is necessary.

The donor site was marked with the patient in a supine position. The width and length of the flap were determined based on the size and location of the tumour, as well as the availability of adipose tissue, which was assessed by a pinch test. Initially, all patients were admitted for an overnight stay postoperatively. After several years of experience, most procedures were performed as day cases unless medical conditions warranted hospitalization.

All patients received a surgical drain, which was removed after 3–8 days. Postoperative care included an initial wound check by a nurse at the breast outpatient clinic, followed by a second visit at 10–14 days for pathology review and wound assessment. Patients were then referred for adjuvant therapy according to multidisciplinary recommendations and recalled for annual follow-up at 11–13 months, which included mammography and clinical examination by a breast surgeon. Subsequent mammography was performed annual for at least five years in accordance with national guidelines.

## Results

A total of 139 patients with breast cancer or DCIS underwent breast-conserving surgery combined with a lateral perforator flap during the study period. Patient and tumour characteristics are summarized in Table 1. The median age was 62 years (range: 33–84 years). The cohort comprised 14% current smokers; (smoking cessation was strongly recommended but not a contraindication for flap reconstruction). Initially, 63% of patients were treated as inpatients, but after six years, the procedure was routinely performed as day surgery. Drains were used in all cases and removed within 3–8 days.

**Table 1:** A. Patient characteristics B. Tumor characteristics.

	No (total=139)	%
Age, years		
-median (range)	62 (33–84)	
BMI		
-median (range)	24 (18–39)	
Smoking		
-non-smoker	104	75
-ex-smoker	15	11
-current smoker	20	14
ASA-class		
−1	67	48
−2	65	47
−3	7	5
Indication		
-tumor size	119	86
-asymmetry	6	4
-reoperation after non-radical surgery	10	7
-others	4	3
Flap performed		
-LICAP	100	72
-LICAP+LTAP	37	27
-TDAP	2	2
No of perforators median (range)	3 (1–5)	
Complications*		
-grade 1	33	24
-grade 2	0	
-grade 3	4	3
-grade 4 and 5	0	
-none	102	73
	No	%
Histological type		
-IDC	93	67
-ILC	23	16
-other IC	6	4
-DCIS + LCIS	16	11
Histological grade		
-I	14	10
-II	74	53
-III	41	30
-unknown	10	7
ER >10%		
-yes	100	72
-no	26	19
-unknown	13	9
PgR >10%		
-yes	79	57
-no	47	34
-unknown	13	9
HER2		
-yes	27	19
-no	99	71
-unknown	13	10
Ki67 >30%		
-yes	42	30
-no	83	60
-unknown	14	10
Lymph node status		
-benign	93	67
-metastasis	44	32
Tumor size, mm		
-median (range)	16 (0–140)	
Tumor extent, mm		
-median	21	
-extent	4–140	
Chemotherapy		
-no	69	50
-adjuvant	43	31
-neoadjuvant	27	19
Radiotherapy		
-no	13	9
-local breast	83	60
-locoregional	43	31
- with boost	16	11

*according to Dindo-Clavien classification.

Neoadjuvant chemotherapy was administered to 27 patients (19%), and 43 patients (31%) received adjuvant chemotherapy. Radiotherapy was given to 91% of patients—(60% to the breast only and 31% locoregionally). For the 13 patients who did not receive radiotherapy, the reasons included complementary mastectomy (n = 5), distant metastasis detected before adjuvant treatment (*n* = 2), pathology-based omission (*n* = 3), patient refusal (*n* = 2), and prior radiotherapy (*n* = 1).

The LICAP flap was most frequently used for surgery (72%). The median tumour size was 16 mm (range: 0–140 mm), and the mean specimen weight was 97 g (range: 12–330 g). The mean percentage of breast volume excised was 22.3% (range: 6.6–55%). The primary indication for flap reconstruction was tumour extent. Flap insertion was performed by turnover in 101 cases, rotation in 30 cases, and undocumented in 8 cases.

Most complications were minor (Clavien-Dindo grade I), and hematoma and infection were the most common. Major complications requiring surgical intervention occurred in 4 patients (2.9%), which were all due to postoperative bleeding. No flap loss occurred. Seven patients had conservatively managed bleeding, 11 had wound infections (which were treated with antibiotics and dressings), and 8 developed minor seromas that resolved spontaneously.

**Fig. 1:**
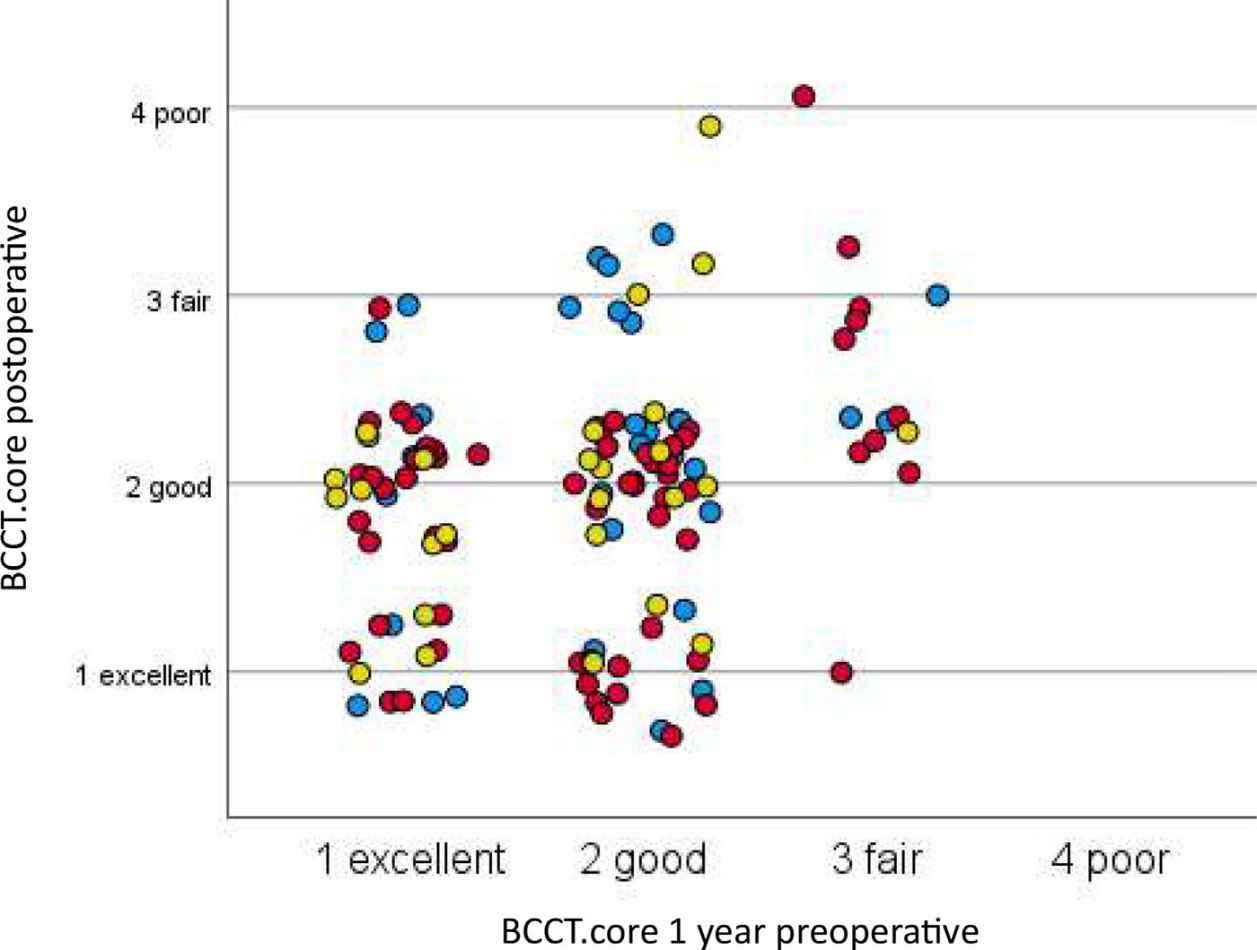
BCCT.core before and after surgery based on the percentage of breast volume excised. <15% breast volume excised 16–30% breast volume excised >31% breast volume excised.

Objective evaluation of the aesthetic outcome using BCCT.core showed that 80% of cases were graded as excellent or good postoperatively; and only 2 patients were graded as poor. Smoking, BMI, and postoperative infection did not significantly affect the BCCT.core scores. Analysis according to the excised breast volume showed that in the group with up to 15% of the breast volume excised, all grades were good or better. In the group with 16–30% excision, 71% of the grades were good or better, and in the group >30% excision, 59% of the grades were good or better (Fig. 1 shows the pre- and postoperative score distribution). The subjective evaluation at the 1-year follow-up showed high satisfaction: the median patient score was 9/10 and the surgeon score was 8/10 on a 10-point Likert scale.

**Figure:**
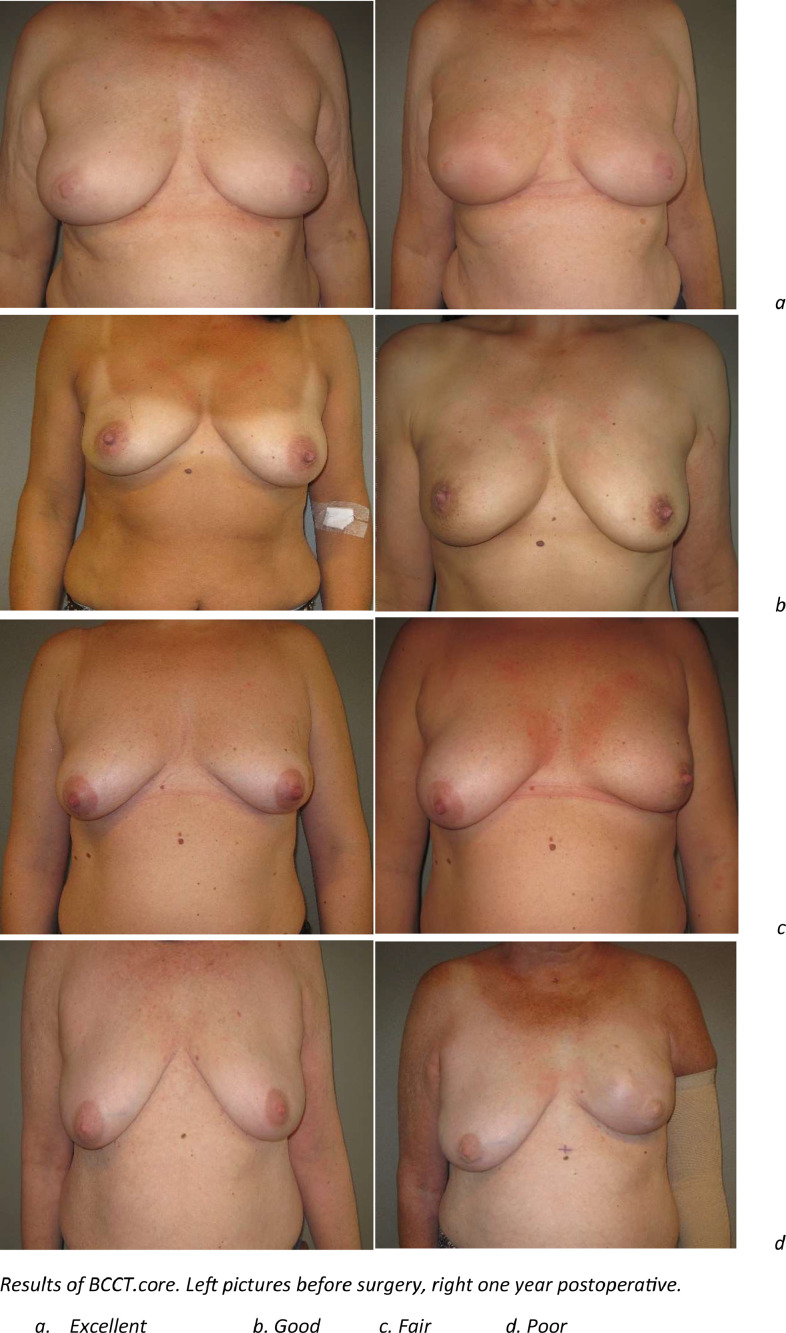

Regarding breast measurements**,** the paired *t*-tests comparing pre- and postoperative breast dimensions (Table 2) showed no significant change in volume. Ptosis and sternal notch–nipple distance decreased slightly but significantly. Contralateral breast measurements showed a slight but significant change in volume. No significant preoperative differences were found between the breasts with cancer and healthy breasts.

**Table 2:** Paired *t*-test on breast dimensions in the cancer breast and the healthy breast comparing pre- and postoperative measures. Preoperative measures are in time of diagnosis and postoperative 1 year after surgery. SNN=sternal notch-nipple.

CANCER BREAST	Mean (sd)	95% CI	p-value
Volume (ml)			
-preoperative	471 (211.5)		
-postoperative	486 (229.7)		
-diff (pre-post)	−15.3 (116.6)	(−36.0, 5.3)	0.144*
SNN (cm)			
-preoperative	24.4 (3.0)		
-postoperative	23.8 (2.9)		
-diff (pre-post)	0.6 (1.2)	(0.4, 0.8)	<0.001*
Ptos (cm)			
-preoperative	2.3 (1.9)		
-postoperative	1.7 (1.6)		
-diff (pre-post)	0.5 (1.0)	(0.4, 0.7)	<0.001*
HEALTHY BREAST	Mean (sd)	95% CI	p-value
Volume (ml)			
-preoperative	482 (217)		
-postoperative	506 (241)		
-diff (pre-post)	−24 (106)	(−43, −5)	0.012*
SNN (cm)			
-preoperative	24.7 (3.1)		
-postoperative	24.6 (2.1)		
-diff (pre-post)	0.1 (1.0)	(−0.8, 0.3)	0.260*
Ptos (cm)			
-preoperative	2.4 (2.0)		
-postoperative	2.4 (1.9)		
-diff (pre-post)	−0.03 (0.8)	(−0.2, 0.1)	0.668*

Regarding oncological outcomes, 7 patients (5%) had local recurrence, and 7 patients (5%) had distant metastasis. Two patients had primary metastases detected postoperatively, and two had concurrent local recurrence and distant metastasis. The median follow-up was 67 months (range: 21–288 months).

## Discussion

Patients with breast cancer are now often long-term survivors and live with the cosmetic outcome of their surgery for many years. The goal of oncoplastic breast surgery is to combine oncological safety with an acceptable aesthetic result. Chest wall perforator flaps offer an alternative to mastectomy for patients with a high tumour-to-breast ratio, and enable breast conservation without compromising radicality.

A unique strength of this study is the availability of pre- and postoperative measurements of both the affected and contralateral breast, including volume, ptosis, and sternal notch–nipple distance. These data allow us to address whether immediate reconstruction with a lateral perforator flap truly preserves the breast. Our findings suggest that breast integrity is maintained, with minimal volume change and only small, but statistically significant reductions in ptosis and sternal notch–nipple distance, which are differences that are not clinically relevant. Variability in measurements may partly reflect inter-observer differences, as multiple surgeons performed the assessments. This variability could also theoretically account for the difference in volume observed in the contralateral unoperated breast.

Cosmetic outcomes were favourable and bbjective evaluation using BCCT.core indicated excellent or good results in 80% of cases, even when >30% of the breast volume was excised. Regression analysis did not identify predictors of poor cosmetic outcome, although the subgroup sizes were small. Subjective ratings by patients and surgeons were high, indicating that minor changes in breast shape are not perceived as important by patients when judging overall cosmesis.

The complication rate was 27%, and the complications were predominantly minor and managed conservatively. Major complications requiring reoperation occurred in only 2.9% of cases. No flap loss occurred. These results align with previously reported complication rates for chest wall perforator flaps (15–36%).^15^, ^16^, ^17^, ^18^ and are lower than those reported for more invasive techniques such as latissimus dorsi flaps.^19^ The multicentre study by Karakatsanis et al.^20^, reported a lower complication rate (8.6%) but shorter follow-up (median 22 months), although our study included only lateral perforator flaps and had a longer median follow-up of 67 months.

Oncological safety is supported by recurrence rates that are consistent with published data. Local recurrence occurred in 5% of patients, and 5% had distant metastasis, which are comparable to rates reported in other oncoplastic series (0–10%).^18^^,^^20^, ^21^, ^22^ These findings suggest that lateral perforator flap reconstruction does not increase the risk of recurrence compared to standard breast-conserving surgery.

The strengths of this study include a relatively large cohort, long follow-up, and detailed bilateral breast measurements. Limitations include its retrospective, single-centre design, which may introduce selection bias and limit generalizability. The absence of a randomized control group precludes direct comparison with other reconstructive options such as latissimus dorsi flaps, implants, or no reconstruction. In summary, our findings confirm that lateral chest wall perforator flaps provide oncologically safe breast conservation with high patient satisfaction and favourable cosmetic outcomes, even in cases requiring extensive tissue excision.

## Funding

The authors have no funding to declare.

## Declaration of competing interest

The authors declare no conflict of interest.
